# The association between serum prolactin levels and live birth rates in non-PCOS patients: A retrospective cohort study

**DOI:** 10.1371/journal.pone.0295071

**Published:** 2023-11-29

**Authors:** Xiaoyuan Xu, Aimin Yang, Yan Han, Wei Wang, Guimin Hao, Na Cui

**Affiliations:** Department of Reproductive Medicine, The Second Hospital of Hebei Medical University, Shijiazhuang, China; China Agricultural University, CHINA

## Abstract

**Background and objectives:**

This paper aimed to analyze the relationship between baseline prolactin (PRL) levels and live birth rates (LBRs) in patients undergoing embryo transfer who did not have polycystic ovarian syndrome (PCOS) using a retrospective design. *Patient(s)*: A total of 20,877 patients who had undergone IVF/intracytoplasmic sperm injection (ICSI) between December 2014 and December 2019.

**Materials and methods:**

We examined the association between PRL concentrations and LBRs using multivariate regression analysis. In addition, a model for nonlinear relationships based on a two-part linear regression was developed.

**Results:**

Following adjustment for confounding factors, multivariate regression analysis confirmed a statistically significant correlation between serum PRL and LBR. Particularly, when blood PRL content was less than 14.8 ng/mL, there exists a positive relation between serum PRL and LBRs. In contrast, once PRL concentrations surpassed the inflection point at 14.8 ng/mL, a meaningful relationship could no longer be inferred between serum PRL and LBR.

**Conclusions:**

Basal serum PRL levels were segmentally connected with LBRs.

## Introduction

Serum prolactin (PRL) is synthesized by PRL-secreting cells. In normal women and patients with hyperprolactinemia, serum PRL molecules are predominantly monomeric (80%) and have the highest biological and immunological activity [1]. Prolactin is crucial for the regulation of reproductive function and PRL enhances the steroidogenic effect of luteinizing hormone in granulosa cells and inhibits the progesterone (P) inactivation by 20alpha-hydroxysteroid dehydrogenase [2]. Serum PRL is known to be usually below 25 ng/L [3]. Hyperprolactinemia is diagnosed when serum PRL is over 25 ng/L. The annual prevalence of hyperprolactinemia in young women has been reported to be 23.9 per 100,000 [4]. It is well-known that hyperprolactinemia directly inhibits the synthesis and release of gonadotropin (Gn), and the frequency and amplitude of pulse secretion are diminished. The positive feedback effect of estrogen disappears, causing impaired follicular development and anovulation. In addition, the relevant vitro research found that serum PRL >100ng/ml and follicular fluid PRL levels are also elevated, which inhibits follicle stimulating hormone (FSH)-induced granulosa cell aromatase activity and estrogen synthesis [5]. The synthesis of progesterone by granulosa luteal cells is dependent on the action of physiological amounts of PRL, and either too high or too low PRL can inhibit the synthesis of progesterone.

In the context of natural pregnancy, high PRL levels appear to be detrimental [6]. Many studies have reported the relationship between transient increase in prolactin levels and pregnancy outcomes although at different time of the stimulation cycle [7–11]. However, there are no studies with a sufficient sample size to elaborate the curvilinear relationship between basal serum PRL levels and live birth rate in IVF/ICSI cycles [7,12]. We aim to explore the effect of basal PRL levels on pregnancy outcomes in women undergoing fresh/frozen embryo transfers and investigate the nonlinear relationship between PRL levels and live births. Is there an appropriate PRL level to improve IVF outcomes?

## Materials and methods

### Study design and population

This research was performed at the Reproductive Medicine Center of the Second Hospital of Hebei Medical University. The data of patients who underwent IVF/ICSI was collected from December 2014 to December 2019.

**Fig 1:**
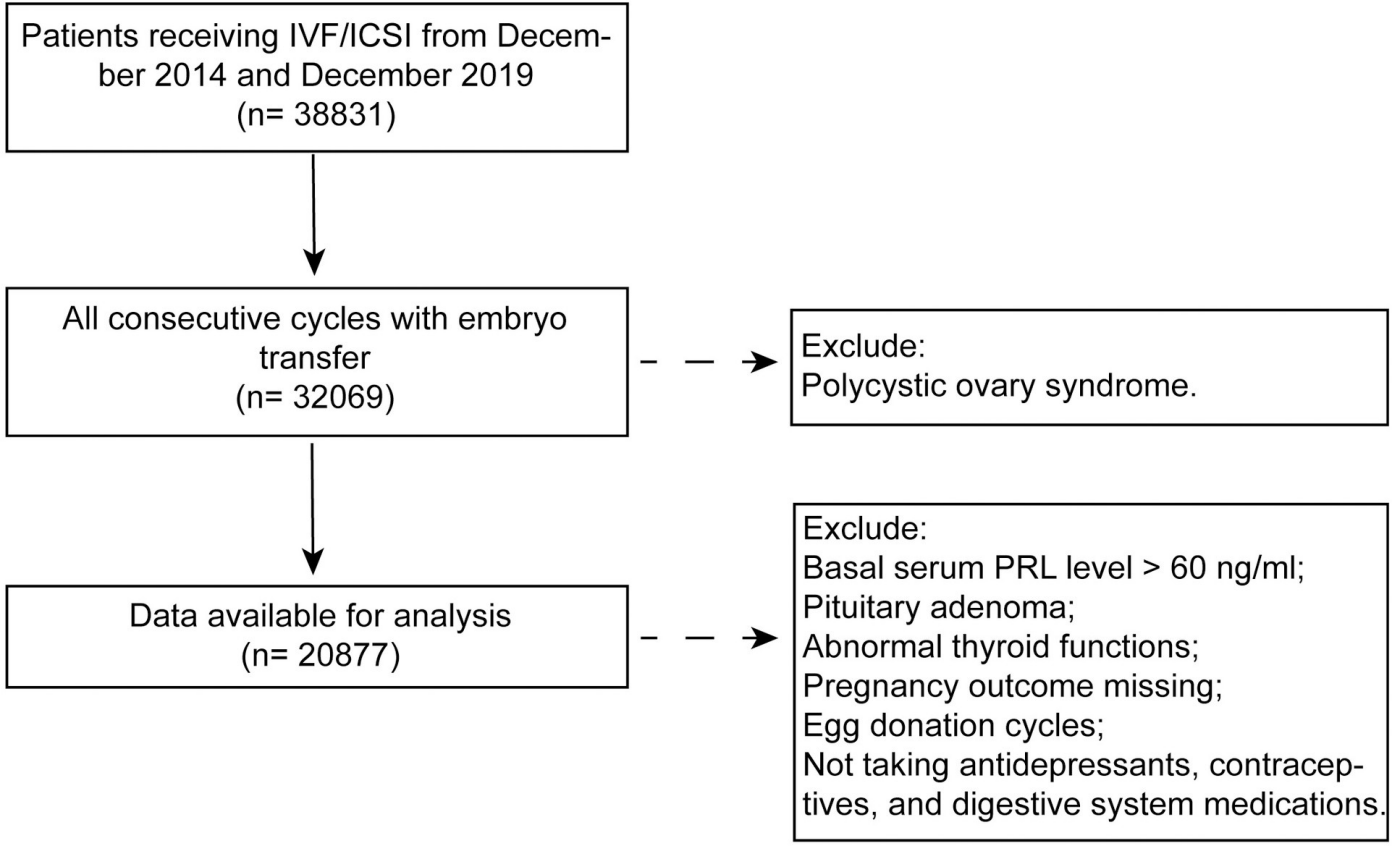
Flow chart for selection of patients from December 2014 to December 2019.

Exclusion criteria included: [1] patients with basal PRL > 60ng/mL, [2] patients with a history of pituitary surgery or radiotherapy for the diagnosis of pituitary adenoma, patients with unsatisfactorily controlled abnormal thyroid function, [3] patients who could not be followed up to outcome, [4] patients with egg donation cycles, [5] patients with a diagnosis of polycystic ovary syndrome, [6] patients not taking antidepressants, contraceptives, and digestive system medications before entering the cycle. A total of 20877 cases were available for research. The screening process is listed in **Fig 1**. The research content was approved by the corresponding Ethics Committee. All subjects agreed to the application of medical records and signed an informed consent form. Infertility patients in our department sign an informed consent form at the time of consultation that their personal medical record information will be used for research studies anonymously, and the patient’s information is entered into the electronic medical record system after the consultation. Our study was a retrospective cohort study and all data were accessed for this research in December 2019.

### Outcome variable

The primary outcome was live birth (no less than one infant born after 24 weeks of gestation) per embryo transfer. Biochemical pregnancy represents serum human chorionic gonadotropin (hCG) level 50mIU/mL 12 days after embryo transfer. It can be regarded as clinical pregnancy if an intrauterine gestational sac is found by vaginal ultrasound.

### Covariates

This paper mainly selects appropriate covariates based on clinical experience and relevant literature. The following covariates were included in the model. Continuous variables included female age (years), infertility duration (years), body mass index (BMI, kg/m2), basal FSH (mIU/ml), antral follicle count (AFC), endometrial thickness on hCG trigger day (mm). Categorical variables included with or without tubal factor, male factor, type of infertility (primary infertility or secondary infertility), number of transferred embryos, and fresh or frozen embryo transfer. At the time of enrolment, patients’ basic demographic data and some personal information were gathered. On the second or third day of menstruation, the baseline hormone levels were assessed. Through medical records, we got information about medications.

### Treatment

For fresh embryo transfer, gonadotropin-releasing hormone agonist (GnRH-a) protocol, ultra-long GnRH-a protocol, and GnRH-antagonist (GnRH-ant) protocol were utilized. GnRH-a protocol: From the middle of the luteal phase, the patient was administered 0.1 mg/day of Decapeptyl. Gonadotropin was provided until the hCG trigger day when down-regulation requirements appeared (luteinizing hormone (LH) < 5 IU/L, estradiol (E2) < 50 pg/L, FSH < 5 IU/L, endometrial thickness < 5 mm, follicle diameter < 5 mm, and no functional ovarian cyst). All of the research objectives were therapied with a depot injection of triptorelin acetate (3.75mg; Ferring Pharmaceuticals, Kiel, Germany), and controlled ovarian stimulation (COS) began 28–35 days after the last treatment. GnRH-ant protocol: GnRH-antagonist (0.25mg/day, Merck Seron, Coinsins, Switzerland) was injected until the hCG trigger day when the follicle measured > 14mm in serum estradiol level was > 400pg/mL.

Individualization of the initial Gn dose is according to age, BMI, and ovarian reserve. Ovarian stimulation was achieved with recombinant/urinary FSH (Gonal-F, Merck Serono, Italy, Urofolitropin for injection, Lizhu Pharmaceutical, China) alone at total doses of 150-300IU/day. Gn can be increased or lowered during COS (serum LH, estradiol, progesterone levels, and diameter of follicles monitored by ultrasound). The trigger day occurred when at least three follicles had a diameter larger than 17 mm with adequate serum hormone levels. It was injected with 8000–10000 IU of hCG (Lishenbao, Livzon Pharmaceutical Co., Ltd.) or 250 g of recombinant human follitropin alfa (MerckSerono S.p.A., Geneva, Switzerland). 16–18 hours after IVF/ICSI insemination, the appearance of the two principal nuclei was evaluated, followed by daily surveillance of embryo development until the third day of oogenesis embryo transfer. Normally fertilized embryos were evaluated using a conventional scoring system [13]. The highest-scoring embryos were transplanted three days after ovulation. The remaining embryos were grown in blastocysts and vitrified for preservation.

For frozen-thawed embryo transfer (FET) the natural cycle and hormone replacement therapy (HRT) were performed for endometrial preparation. Luteal support was provided by oral dydrogesterone (10mg; Duphaston, Abbott, OLST, Netherlands) twice a day for natural and letrozole-induced ovulation protocols. To provide luteal support, the HRT program included oral dydrogesterone 10 mg three times daily and vaginal progesterone gel (90mg; Crinone, Merck Serono, Watford, UK) once daily. If pregnancy was detected, luteal support was maintained for the first eight weeks of gestation. Embryos with the highest morphological scores were favored for transfer at early cycles of embryo transfer.

### Statistical methods

The measurement data of normal distribution are described by means of mean ± standard deviation (SD); non-normal distribution is described based on quantile and percentage. ANOVA (normally distributed continuous variables), chi-square (categorical variables), and Kruskal-Wallis (non-normally distributed continuous variables) tests were conducted for differences. Based on the test results, it was judged whether there were significant statistical differences in the baseline characteristics of various pregnancy outcomes. Univariate analysis was conducted on the correlation between covariates and LBR to determine the variables that have a significant impact on LBRs.

In multivariate logistic analysis, three models were established. The crude model was estimated without adjustment. The adjusted model I was determined by adjusting the factors of age and BMI. Then the following variables were adjusted in the adjusted model II: female age, infertility duration, BMI, basal FSH, tubal factor, male factor, AFC, endometrial thickness on hCG trigger day, infertility type, number of transferred embryos, fresh or frozen embryo transfer. Then, based on stratification, research and discussion are carried out, and regression analysis is carried out for each covariate. Interaction analysis was performed for female age (≤35 and >35 years), BMI (≤24 and >24kg/m2), infertility duration (≤1 and >1 year), basal FSH (≤10 and >10mIU/ml), AFC (≤12 and >12), endometrial thickness on hCG trigger day (≤7 and >7 mm), with or without tubal factor, with or without male factor, number of transferred embryos (1,2 and 3), method of fertilization (IVF, ICSI and IVF&ICSI) and fresh or frozen embryo transfer. Additionally, serum PRL level was transformed into a categorical variable to check the possibility of curvilinear association.

In the research of data fitting, binary logical regression, and smooth curve model are used to determine the correlation between PRL level and LBRs variable based on the results. The minimum GCV method is used to analyze and determine the degree of freedom, which is 2.2715 in this paper. In the process of correlation fitting, two piecewise binary logistic regression models are used, and the best-fitting equation is selected for the test. In the fitting process, to facilitate processing, the inflection point is moved along a predetermined interval, and the maximum likelihood inflection point of the established model is determined accordingly. The basis of statistical difference was *P*< 0.05. Statistical software packages R (http://www.R-project.org, The R Foundation) and EmpowerStats software (http://www.mpowerstats.com, X&Y Solution, Inc., Boston, MA) were used in the statistical analysis process.

## Results

### Patient characteristics

We studied 20877 patients of received treatment from December 2014 to December 2019, of whom 12111 had live births and 8766 had no live births. The baseline feature and embryo parameters were listed in Table 1. Compared to the patients with live births, patients without live births were older (p <0.001), had a lower AFC (p <0.001), and thinner endometrium on hCG trigger day (p <0.001), had higher BMI (p <0.01), had lower prolactin level (p <0.001). Patients with live births spent less Gn medicine (2284.67 ± 1167.86 vs. 2339.59 ± 1719.64, p <0.05), retrieved more oocytes (10.63 ± 4.91 vs. 9.39 ± 5.30, p <0.001), although they had fewer days of Gn application (8.87 ± 4.48 vs. 8.60 ± 4.61, p <0.001).

**Table 1:** Demographic characteristics of the participants stratified by outcome.

Demographic characteristics	No live birth	Live birth	*P* value
N	12111	8766	
Female age (y)	31.15 ± 4.98	29.81 ± 4.05	<0.001
Male age (y)	31.94 ± 5.53	30.68 ± 4.58	<0.001
Infertility duration (y)	3.0 (2.0–5.3)	3.0 (2.0–5.0)	<0.001
BMI (kg/m2)	23.25 ± 3.58	23.10 ± 3.45	<0.01
Basal FSH (mIU/ml)	7.88 ± 3.95	7.53 ± 3.33	<0.001
Basal E2 (pg/ml)	30.00 (14.58–47.00)	30.00 (14.99–46.57)	0.328
Basal P (ng/ml)	0.61 (0.37–0.92)	0.62 (0.37–0.93)	0.301
Basal PRL (ng/ml)	13.63 ± 6.94	13.96 ± 6.91	<0.001
Basal LH (mIU/ml)	4.26 (3.10–5.85)	4.30 (3.16–5.83)	0.942
AFC	12.49 ± 5.59	13.44 ± 5.38	<0.001
Endometrial thickness on hCG trigger day (mm)	10.20 ± 1.96	10.49 ± 1.98	<0.001
Infertility type			<0.001
Primary infertility	6566 (54.2%)	5033 (57.4%)	
Secondary infertility	5545 (45.8%)	3733 (42.6%)	
Fresh or frozen embryo transfer			0.096
Embryo transfer	6747 (55.7%)	4985 (56.9%)	
Frozen-thrawed embryo transfer	5364 (44.3%)	1629 (43.1%)	
Gn total dose (IU)	2339.59 ± 1719.64	2284.67 ± 1167.86	<0.05
Gn duration (day)	8.60 ± 4.61	8.87 ± 4.48	<0.001
Method of fertilization			0.073
IVF	5374 (79.7%)	3907 (78.4%)	
ICSI	1353 (20.0%)	1052 (21.1%)	
IVF&ICSI	18 (0.3%)	23 (0.5%)	
No. of oocytes retrieved	9.39 ± 5.30	10.63 ± 4.91	<0.001
Embryo stage			0.434
Cleavage stage	11461 (96.3%)	5694 (96.1%)	
Blastocyst stage	439 (3.7%)	336 (3.9%)	
No. of transferred embryos			<0.001
1	1214 (10.0%)	358 (4.1%)	
2	9558 (78.9%)	7618 (86.9%)	
3	1339 (11.1%)	790 (9.0%)	

### Outcomes of univariable analysis

Univariate analyses were carried out to assess the influence of each variable on LBR (Table 2). The outcome of univariable analysis showed significant impact of PRL levels on LBR [OR 1.01 (1.00, 1.01); p <0.001]. Patients with male factor significantly increased LBR as compared with patients without male factor [OR 1.20 (1.12, 1.28); p <0.001]. AFC [1.01 (1.00, 1.01); p <0.001], Gn total dose [1.02 (1.00, 1.04); p <0.05], and endometrial thickness on hCG trigger day [OR 1.08 (1.06, 1.09); p <0.001] had a positive impact on LBR. Moreover, 2 [OR 2.70 (2.39, 3.05); p <0.001] or 3 [OR 2.00 (1.73, 2.32); p <0.001] transferred embryos were positively associated with live births compared to patients with 1 embryo transfer.

**Table 2:** Univariate analysis for live birth rate.

	Statistics	OR (95%Cl)	*P* value
Female age (y)	30.59 ± 4.66	0.94 (0.93, 0.94)	<0.001
Infertility duration (y)	3.94 ± 3.01	0.96 (0.95, 0.97)	<0.001
BMI (kg/m^2^)	23.19 ± 3.53	0.99 (0.98, 1.00)	<0.01
Basal FSH (mIU/ml)	7.73 ± 3.71	0.97 (0.96, 0.98)	<0.001
Basal E2 (pg/ml)	37.08 ± 46.10	1.000 (1.000, 1.000)	0.329
Basal P (ng/ml)	1.00 ± 6.09	1.00 (0.99, 1.00)	0.322
Basal LH (mIU/ml)	5.09 ± 3.95	1.00 (0.99, 1.01)	0.942
Basal PRL (ng/ml)	13.77 ± 6.93	1.01 (1.00, 1.01)	<0.001
AFC	12.90 ± 5.52	1.01 (1.00, 1.01)	<0.001
Male factor			
Without male factor	16072 (77.8%)	Reference	
With male factor	4594 (22.2%)	1.20 (1.12, 1.28)	<0.001
Tubal factor			
Without tubal factor	3904 (22.4%)	Reference	
With tubal factor	13495 (77.6%)	0.72 (0.67, 0.78)	<0.001
Fresh or frozen embryo transfer			
Fresh embryo transfer	11732 (56.2%)	Reference	
Frozen-thawed embryo transfer	9145 (43.8%)	0.95 (0.90, 1.01)	0.096
Endometrial thickness on hCG trigger day (mm)	10.32 ± 1.97	1.08 (1.06, 1.09)	<0.001
Infertility type			
Primary infertility	11599 (55.6%)	Reference	
Secondary infertility	9278 (44.4%)	0.88 (0.83, 0.93)	<0.001
Gn total dose (IU)	10.81 ± 2.42	1.02 (1.00, 1.04)	<0.05
Gn duration (day)	2635.52 ± 1427.57	1.000 (1.000, 1.000)	<0.001
Method of fertilization			
IVF	9281 (79.1%)	Reference	
ICSI	2405 (10.5%)	1.07 (0.98, 1.17)	0.146
IVF&ICSI	41 (0.4%)	1.76 (0.95, 3.26)	0.074
No. of transferred embryos			
1	1572 (7.5%)	Reference	
2	17176 (82.3%)	2.70 (2.39, 3.05)	<0.001
3	2129 (10.2%)	2.00 (1.73, 2.32)	<0.001

The results showed the LBR decreased with the increasing BMI [OR 0.99 (0.98, 1.00); p <0.01], age [OR 0.94 (0.93, 0.94); p <0.001], basal FSH levels [OR 0.97 (0.96, 0.98); p <0.001], infertility duration [OR 0.96 (0.95, 0.97); p <0.001]. Patients with secondary infertility [OR 0.88 (0.83, 0.93); p <0.001] and tubal infertility [OR 0.72 (0.67, 0.78); p <0.001] had lower LBR when compared with patients with primary infertility and without tubal factors.

### Outcomes of multivariable analysis

To examine the association between serum PRL levels and LBR, three models were built using logistic regression. Table 3 displays the odds ratio (OR) and 95% confidence interval (CI). A positive correlation between PRL and LBR was discovered in the crude model [OR 1.07 (1.03, 1.11); p <0.001]. After accounting for female age and BMI in model I, the relationships in regression analyses did not differ significantly from those in the crude model [OR 1.03 (0.99, 1.08); p = 0.107]. Taking all confounding factors in to account [female age, BMI, infertility duration, basal FSH, AFC, infertility type, fresh or frozen embryo transfer, tubal factor, male factor, number of transferred embryos, and endometrial thickness on hCG trigger day], the results of multivariable regression analysis failed to show a significantly positive effect between PRL levels and LBR [OR 1.05 (0.99,1.10); p = 0.087].

**Table 3:** Multivariable regression analysis examining the association between PRL level and LBR.

LBR	Crude model^a^		Model I^b^		Model II^c^	
	OR (95%CI)	*P* value	OR (95%CI)	*P* value	OR (95%CI)	*P* value
Basal PRL (ng/ml)	1.07 (1.03, 1.11)	<0.001	1.03 (0.99, 1.08)	0.107	1.05 (0.99,1.10)	0.087
Patients were equally divided into three groups according to PRL levels						
Low	Reference		Reference		Reference	
Intermediate	1.11 (1.03, 1.18)	<0.01	1.07 (1.00, 1.15)	0.053	1.10 (1.01, 1.20)	<0.05
High	1.14 (1.07, 1.22)	<0.001	1.08 (1.00, 1.15)	<0.05	1.11 (1.02, 1.21)	<0.05
Three groups treated as continuous variables	1.07 (1.03, 1.11)	<0.001	1.04 (1.00, 1.07)	<0.05	1.05 (1.01, 1.10)	<0.05

^a^ No adjustments for covariates.

^b^ Adjusted for female age and BMI.

^c^ Adjusted for all covariables in model I plus infertility duration, basal FSH, AFC, infertility type, fresh or frozen embryo transfer, tubal factor, male factor, number of transferred embryos, and endometrial thickness on hCG trigger day.

**Fig 2:**
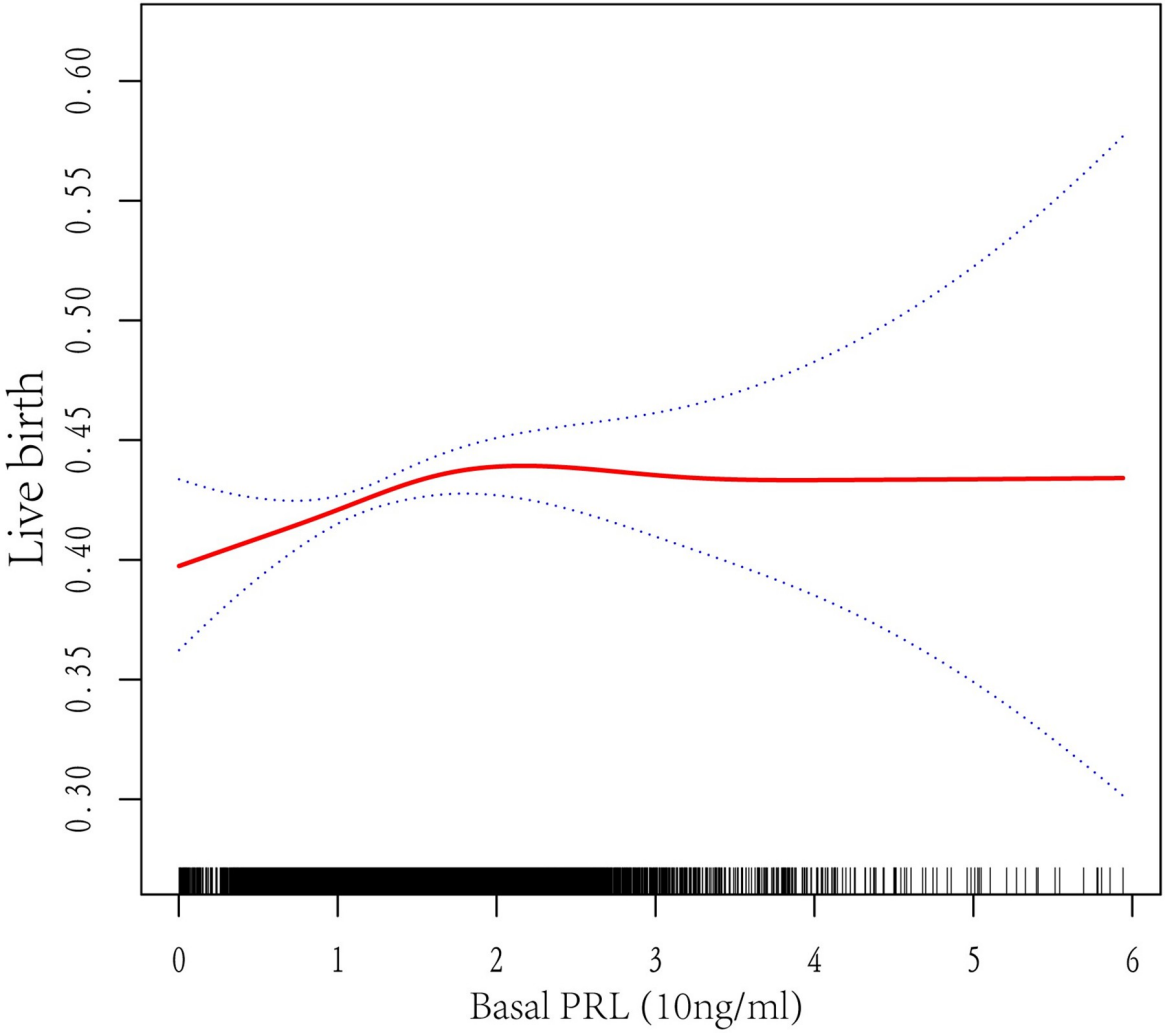
Association between live birth and basal serum PRL level. A threshold, nonlinear association between live birth and basal serum PRL level was found in a generalized additive model (GAM). Solid red line represents the smooth curve fit between these two variables. Blue lines represent the 95% of confidence interval from the fit.

By using binary logistic regression and the penalty curve technique, it was possible to identify the curvilinear link between basal PRL levels and LBR. After taking into account confounders, including baseline demographic measures and intervention techniques, there exists a curvilinear relation between serum PRL levels and LBRs (Fig 2). A recursive method determined the inflection point to be 14.8 ng/mL PRL (Table 4). When serum PRL > 14.8 ng/mL, there was no significant between LBRs and PRL levels [OR 0.98 (0.90, 1.06); p = 0.56]. However, LBRs increased significantly by 18% for every 10 ng/mL PRL when serum PRL < 14.8 ng/mL [OR 1.18 (1.04, 1.33); p <0.01].

**Table 4:** The result of two-piecewise linear regression model.

Models	Adjusted OR (95%Cl)	*P* value
Model I^a^		
**One line slope**	1.05 (0.99, 1.10)	0.087
Model II^b^		
** Inflection point**	1.48	
** < 1.479**	1.18 (1.04, 1.33)	<0.01
** > 1.479**	0.98 (0.90, 1.06)	0.56
LRT test^c^	0.033	

^a^ Linear analysis.

^b^ Nonlinear analysis.

^c^ LRT test, logarithmic likelihood ratio test (p value < 0.05 indicates that Model II is significantly different from Model I, which indicates a nonlinear relationship). Adjusted for female age, infertility duration, BMI, infertility duration, basal FSH, AFC, infertility type, fresh or frozen embryo transfer, tubal factor, male factor, number of transferred embryos, and endometrial thickness on hCG trigger day.

### Outcomes of stratification and interaction analyses

The impact of PRL levels on LBR was further analyzed with multivariable regression model in several subgroups. The results suggested that PRL levels did not impact LBR in all subgroups. Interactions between the stratification factors and PRL levels were tested, and the results showed that the stratification factors failed to have the interaction effect with PRL levels on LBR (P > 0.05 for all comparisons) (S1 Table).

## Discussion

Our findings suggested a threshold saturation effect of basal PRL levels (≤ 60 ng/ml) with LBR in non-PCOS patients. When the basal serum PRL levels were lower than 14.8 ng/ml, LBR enhanced about every 10 ng/ml in PRL. When basal PRL levels were higher than 14.8 ng/ml, there was no obvious association between the two.

High levels of PRL have positive effects, which are mainly reflected in the luteal function. In the absence of PRL, women and rats will lack normal luteal function and become infertile [14]. Progesterone replacement can rescue normal implantation of the embryo in early Chinese pregnancies [15]. However, in IVF/ICSI, luteal stage support is given with enough progesterone to ensure. Is it then needed to suppress basal state PRL levels? Are basal state PRL levels related to pregnancy outcome? Does prolactin affect pregnancy outcomes in ways other than luteal function?

Many scholars have researched the relationship between PRL and embryo quality in ICSI. Mendaza et al. found that higher concentrations of PRL in follicles were prone to cause normal fertilization [16]. Embryo quality, fertilization rate, and mean number of embryos transferred were higher in the untreated group with high PRL compared to the treated group with medication such as dopamine [17]. Some researchers have even attempted to improve IVF with a history of repeated fertilization failures until the rFSH start date produces a rebound in PRL. The results reflect a fact that the pregnancy rate and eugenics rate did improve compared to the control group [18]. This suggests a role for PRL in oocyte maturation and embryo development. Recent studies have found a positive association between higher-quality embryos and PRL levels before oocytes retrieval, but no appropriate inflection point has been studied [7,12]. On top of this, our study illustrated the saturation of its beneficial effect and proper inflection point.

PRL has also been shown to be involved in the regulation of mammalian oocytes in the following ways. It is known that messenger RNAs for the different isoforms of PRL receptors have been identified in oocytes and oocytes [19]. The cyclic variation in ovarian mRNA encoding PRL receptors during the motile cycle implies that the PRL receptor is also involved in multiple functions of granulosa cells and oocytes [20]. During human embryo development, the embryo expresses PRLR at the mulberry and blastocyst stages. PRLR signaling during trophectoderm growth stimulates blastocyst adhesion [21]. It also has metabolic, transcriptomic, and proteomic functions at the stalled stage (a reversible state of arrested blastocyst development), similar to pluripotent stem cells, affecting oocyte maturation and neurological development. [22]. In contrast, PRL receptor gene deficient mice can only produce eggs with intact germinal foam [23]. However when PRL was added to the in vitro fertilization culture system, favorable effects could be observed [24]. Later studies also confirmed that PRL can significantly increase oocyte maturation rate and early embryonic development [25]. As for animal experiments, Kiapekou et al. found that the positive effects of different PRL isoforms on growth and development may be manifested at various stages of mouse preimplantation embryos [26]. The positive effect of PRL on oocyte potential was also found in bovine oocytes through oocytes containing PRL receptors [19]. The signaling pathway of PRL can be manifested in several aspects. Several studies have found beneficial effects on oocyte developmental capacity through oocytes containing PRL receptors [19,27]. Additionally, PRL slows down the aging process of mature bovine oocytes in vitro. This may be accomplished by lowering the activation rate of denuded oocytes (DO), as well as by activating MAP kinases, which can keep oocyte activity [28]. Recent research has revealed that PRL promotes progesterone synthesis based on enhancing StAR and 3HSD expression and that PRL inhibits FSH-induced estradiol production by decreasing aromatase expression. Notably, the mechanism through which PRL modifies granulosa steroidogenesis caused by FSH is believed to involve the ERK1/ERK2 pathway rather than the cAMP-protein kinase A [23]. In addition to activating STAT dimerization and nuclear translocation [29,30], PRL activates the Src family of tyrosine kinases, phosphatidylinositol 3-kinase (P13K) [31,32]. Interestingly, PRL is found to be involved in embryo implantation via BRCA1. Recent studies have found that although PRL promotes BRCA1 protein levels in trophectoderm, PRL treatment alone does not improve blastocyst implantation, probably because BRCA1 is not necessary for the attachment response in mouse blastocysts. However, combined supplementation with PRL, 4-hydroxyestradiol can improve relevant implantation potential, which is associated with embryo transfer, and this deserves further exploration [33].

The relationship between PRL levels and LBRs in this study reflects that when PRL levels were very high, there was no beneficial relationship. It has been reported that high PRL levels will lead to no chance of conception and inhibit granulosa cell luteinization and steroidogenesis [34]. Results from animal model studies suggest that hyperprolactinemia inhibits the pulsatility of gonadotropin-releasing hormone by reducing kisspeptin input. kisspeptin is considered to be the primary control point for reproduction. Clinical treatment with dopaminergic agonists such as bromocriptine is often applied to reverse the hypogonadal state in hyperprolactinemia, which is effective in 80–90% of hyperprolactinemic patients. elevated PRL levels in serum and follicular fluid during ovulation may inhibit oocyte fertilization and oogenesis [35].

However, due to the lack of a known etiology, some cases of hyperprolactinemia are categorized as "idiopathic" [36]. A massive immune complex of PRL and IgG (macroprolactin) is formed when anti-PRL autoantibodies attach to PRL, which has a relative molecular mass of 23 kDa. This immune complex tends to raise blood levels of PRL [37]. The incidence of macroprolactins, which are circulating autoantibodies against prolactin and immunoglobulin G that are six to seven times more massive than the natural prolactin molecule, is between 10 to 25 percent in people with hyperprolactinemia [38]. Macroprolactins are considered to be biologically inactive because their large size prevents them from binding to PRLR. This form of prolactin molecules is not harmful and does not require treatment. Therefore, asymptomatic patients with recurrent elevated prolactin must consider the possibility of large molecules of hyperprolactinemia. The use of bromocriptine in all patients with measured elevated prolactin may lead to over-medication of some patients. This study did not distinguish between types of prolactin. Further studies could perform stratification of prolactin, such as the polyethylene glycol method [39], to exclude patients with large macromolecular hyperprolactinemia.

Our work has some noteworthy advantages. First, the sample size is sufficient and statistical meaning is ensured. Secondly, this paper regulated many variables to make the results more reliable. Third, we used the correct statistical methods to ensure the stability of the data structure. Finally, our study is a retrospective cohort study.

This research still has some limitations, as shown in the following aspects. First, due to retrospective research, we can’t analyze other confounding factors that might affect pregnancy outcomes, such as AMH, smoking, and nutritional supplementation. Second, we stratified fresh and frozen-thawed embryo transfers but did not stratify the protocols in fresh/frozen cycles, which may be a potential confounding factor. However, we controlled for confounding factors by multivariate logistic regression and performed stratified research to ensure the stability of the results. In addition, we excluded patients with PCOS because the PRL levels of PCOS patients can be affected by their hyperandrogenism or ovulation disorders, which may interfere with our conclusions [40]. Finally, these data are from a single reproductive medicine center and require validation in a multicenter study.

In conclusion, the basal PRL level seems to exist segmental relation with the LBR. Further multicenter randomized controlled trials are needed to confirm the impact of basal PRL levels on pregnancy outcomes. These studies will help predict patient pregnancy outcomes and personalize treatment.

## Conclusion

Based on our research, the PRL level appears to have a segmental relationship with the LBR. Further prospective studies are required to explore the possible mechanisms and to consolidate the association between serum PRL levels and LBR.

## Supporting information

S1 Checklist STROBE statement—Checklist of items that should be included in reports of observational studies. (PDF)

S1 File (TXT)

S1 Table Stratification analysis in different subgroups. (DOCX)
