# Supplementary material for: The association between serum prolactin levels and live birth rates in non-PCOS patients: A retrospective cohort study
Source: PLoS One. 2023 Nov 29;18(11):e0295071. doi: 10.1371/journal.pone.0295071 (PMC10686428; doi:10.1371/journal.pone.0295071)
Supplement: S1 Table — (DOCX) [file pone.0295071.s003.docx]

**S1 Table****. Stratification analysis in different subgroups.**

| **Stratification characteristics** | **N** | **LBR** | | ***P* for interaction** |
| --- | --- | --- | --- | --- |
|  |  | **Adjusted OR (95%Cl)** | ***P* value** |  |
| Female age |  |  |  | 0.421 |
| ≤35 | 17785 | 1.027 (1.006, 1.051) | <0.01 |  |
| >35 | 3091 | 1.012 (0.950, 1.093) | 0.346 |  |
| BMI (kg/m^2^) |  |  |  | 0.086 |
| ≤24 | 13130 | 1.021 (0.957, 1.089) | 0.053 |  |
| >24 | 7467 | 1.122 (1.028, 1.225) | <0.01 |  |
| Basal FSH (ng/ml) |  |  |  | 0.118 |
| ≤10 | 18263 | 1.067 (1.010, 1.128) | <0.05 |  |
| >10 | 2588 | 0.933 (0.795, 1.095) | 0.394 |  |
| AFC |  |  |  | 0.932 |
| ≤12 | 9139 | 1.055 (0.982, 1.133) | 0.142 |  |
| >12 | 7911 | 1.050 (0.972, 1.133) | 0.214 |  |
| Tubal factor |  |  |  | 0.367 |
| Without tubal factor | 3904 | 1.010 (0.910, 1.121) | 0.850 |  |
| With tubal factor | 13495 | 1.068 (1.005, 1.134) | <0.05 |  |
| Male factor |  |  |  | 0.052 |
| Without male factor | 16072 | 1.069 (1.012, 1.129) | <0.05 |  |
| With male factor | 4594 | 0.884 (0.734, 1.065) | 0.194 |  |
| Endometrium thickness on hCG trigger day (mm) |  |  |  | 0.381 |
| ≤7 | 764 | 0.927 (0.694, 1.238) | 0.609 |  |
| >7 | 19216 | 1.056 (1.001, 1.114) | <0.05 |  |
| Infertility type |  |  |  | 0.474 |
| Primary infertility | 11599 | 1.036 (0.968, 1.110) | 0.307 |  |
| Secondary infertility | 9278 | 1.077 (0.994, 1.168) | 0.071 |  |
| Fresh or frozen embryo transfer |  |  |  | 0.677 |
| Fresh embryo tranfer | 11732 | 1.063 (0.993, 1.139) | 0.081 |  |
| Fronzen-thawed embryo transfer | 9145 | 1.040 (0.960, 1.127) | 0.337 |  |

The following factors, except the stratification factor itself, were adjusted in the multivariable analysis: female age, BMI, infertility type, infertility duration, number of transferred embryos, basal FSH, AFC, fresh or frozen embryo transfer, endometrial thickness on hCG trigger day.
